# Safety and Immunogenicity Analysis of a Newcastle Disease Virus (NDV-HXP-S) Expressing the Spike Protein of SARS-CoV-2 in Sprague Dawley Rats

**DOI:** 10.3389/fimmu.2021.791764

**Published:** 2021-11-18

**Authors:** Johnstone Tcheou, Ariel Raskin, Gagandeep Singh, Hisaaki Kawabata, Dominika Bielak, Weina Sun, Irene González-Domínguez, D Noah Sather, Adolfo García-Sastre, Peter Palese, Florian Krammer, Juan Manuel Carreño

**Affiliations:** ^1^ Department of Microbiology, Icahn School of Medicine at Mount Sinai, New York, NY, United States; ^2^ Center for Global Infectious Disease Research, Seattle Children’s Research Institute, Seattle, WA, United States; ^3^ Department of Pediatrics, University of Washington, Seattle, WA, United States; ^4^ Department of Global Health, University of Washington, Seattle, WA, United States; ^5^ Department of Medicine, Division of Infectious Diseases, Icahn School of Medicine at Mount Sinai, New York, NY, United States; ^6^ Global Health and Emerging Pathogens Institute, Icahn School of Medicine at Mount Sinai, New York, NY, United States; ^7^ The Tisch Cancer Institute, Icahn School of Medicine at Mount Sinai, New York, NY, United States; ^8^ Department of Pathology, Molecular and Cell-Based Medicine, Icahn School of Medicine at Mount Sinai, New York, NY, United States

**Keywords:** COVID-19, SARS-CoV-2, newcastle disease virus, vaccine, immunogenicity, safety, rat model

## Abstract

Despite global vaccination efforts, severe acute respiratory syndrome coronavirus 2 (SARS-CoV-2) continues to evolve and spread globally. Relatively high vaccination rates have been achieved in most regions of the United States and several countries worldwide. However, access to vaccines in low- and mid-income countries (LMICs) is still suboptimal. Second generation vaccines that are universally affordable and induce systemic and mucosal immunity are needed. Here we performed an extended safety and immunogenicity analysis of a second-generation SARS-CoV-2 vaccine consisting of a live Newcastle disease virus vector expressing a pre-fusion stabilized version of the spike protein (NDV-HXP-S) administered intranasally (IN), intramuscularly (IM), or IN followed by IM in Sprague Dawley rats. Local reactogenicity, systemic toxicity, and post-mortem histopathology were assessed after the vaccine administration, with no indication of severe local or systemic reactions. Immunogenicity studies showed that the three vaccination regimens tested elicited high antibody titers against the wild type SARS-CoV-2 spike protein and the NDV vector. Moreover, high antibody titers were induced against the spike of B.1.1.7 (alpha), B.1.351 (beta) and B.1.617.2 (delta) variants of concern (VOCs). Importantly, robust levels of serum antibodies with neutralizing activity against the authentic SARS-CoV-2 USA‐WA1/2020 isolate were detected after the boost. Overall, our study expands the pre-clinical safety and immunogenicity characterization of NDV-HXP-S and reinforces previous findings in other animal models about its high immunogenicity. Clinical testing of this vaccination approach is ongoing in different countries including Thailand, Vietnam, Brazil and Mexico.

## Introduction

The pandemic caused by severe acute respiratory syndrome coronavirus 2 (SARS-CoV-2) has afflicted over 250 million people worldwide and has caused the death of at least 5 million people (WHO, 2021). To mitigate the burden caused by this virus, diverse countermeasures have been established worldwide, including social distancing, use of face masks, and more recently the rollout of different vaccines (1). Currently, in the United States three vaccines have been authorized to prevent the coronavirus disease 2019 (COVID-19): two mRNA-based approaches, including BNT162b2 (from Pfizer) and mRNA-1273 (from Moderna), and the viral vector-based approach JNJ-78436735 (from Johnson & Johnson) (2–4). Recently, BNT162b2 was fully licensed by the U.S. Food and Drug Administration as the first vaccine to prevent COVID-19 (5). The generation of these vaccines in such a record time and their rollout in the population - with an initial Emergency Use Authorization (EUA) - represents a milestone for vaccine development and science in general. Overall, COVID-19 vaccines have proven to be effective and display a robust safety profile, with very few people experiencing severe adverse effects (6). Moreover, as stated by the World Health Organization (WHO), the potential benefits of vaccination outweigh potential risks (7). Importantly, vaccination has been directly associated with prevention of viral infection (8, 9), reduced virus transmission (10), attenuation of severe disease (11, 12), and reduced viral load during breakthrough infections (13). Mechanisms of vaccine-induced protection involve the induction of neutralizing antibodies, mostly directed against the viral spike protein (14, 15), as well as cellular responses (16, 17).

While there are a handful of vaccines widely available in the United States and many regions worldwide, the number of vaccine doses available and the vaccination rates in places with limited resources is still suboptimal. Particularly, large parts of Africa, Asia, and some countries of Latin-America display poor vaccination rates (18, 19). Moreover, specific populations, such as immunocompromised or transiently immunosuppressed individuals, might require additional vaccine doses to elicit robust immune responses (20–22). Even healthy individuals at high risk, such as healthcare workers and elderly might be in need of multiple vaccine doses (23, 24). Importantly, the current vaccines available display limited induction of mucosal immunity that prevents infection in the upper respiratory tract, as compared to natural infection (25). Hence, expansion of the existing vaccination platforms and development of novel vaccination approaches that elicit robust immune responses - especially in the upper respiratory tract - is imperative.

Different COVID-19 vaccines are being currently developed, including: a) additional/alternative mRNA vaccines, b) additional whole virus inactivated vaccines, c) live virus attenuated vaccines, d) recombinant protein (RBD or spike) based vaccines, e) virus like particle (VLP) based vaccines, f) DNA-based vaccines, and g) additional vector-based vaccines. Within this last category, replication competent, replication incompetent and inactivated vector-based vaccines are in development and replication incompetent vectors are authorized already (26). Here we report the safety and immunogenicity profile of a vaccine candidate (NDV-HXP-S) based on the Newcastle Disease Virus (NDV) - LaSota strain - expressing a modified version of the spike protein on its surface. The altered spike lacks the polybasic cleavage site and includes six prolines to maintain the pre-fusion conformation of the protein. The transmembrane and C-terminal domains of the spike protein were swapped with the corresponding domains of the NDV fusion protein to allow the integration of the spike into NDV particles. We evaluated the local reactogenicity and the systemic toxicity, including post-mortem histopathology, after administration of NDV-HXP-S to Sprague Dawley rats. All the vaccination regimens tested elicited high antibody titers against the spike protein of wild type SARS-CoV-2 and different variants of concern (VOCs). Importantly, vaccination elicited high levels of neutralizing antibodies against the original SARS-CoV-2 USA‐WA1/2020 isolate. In light of the existing pre-clinical data in other animal models (27–30), and the current testing of this vaccination platform in humans - ongoing clinical trials in Thailand (NCT04764422), Vietnam (NCT04830800), Mexico (NCT04871737), and Brazil (NCT04993209) -, our study reinforces and expands the safety profile and the immunogenicity characteristics of NDV-HXP-S.

## Materials and Methods

### Experimental Vaccine and Recombinant Proteins

Live NDV-HXP-S was produced under current Good Manufacturing Practices (cGMP) regulations in the Vaccine and Cell Therapy Laboratory (VCTL) facility (Icahn School of Medicine at Mount Sinai) as previously described (29). Wild type spike of SARS-CoV-2 as well as B.1.1.7, B.1.351 and B.1.617.2 SARS-CoV-2 were generated and expressed in 293F cells as previously described (31). Briefly, for recombinant protein expression, the mammalian-cell codon-optimized nucleotide sequence of a soluble spike protein (amino acids 1-1,213) lacking the polybasic cleavage site, carrying two stabilizing mutations (K986P and V987P), a signal peptide, and at the C-terminus a thrombin cleavage site, a T4 fold-on trimerization domain, and a hexahistidine tag was cloned into the mammalian expression vector pCAGGS. The expression plasmids used are available at BEI Resources repository (https://www.beiresources.org/). Protein was purified using gravity flow purification with Ni-nitrilotriacetic acid (NTA) agarose (Qiagen) and concentrated and buffer exchanged in Amicon centrifugal units (EMD Millipore). The purified recombinant proteins were analyzed *via* reducing sodium dodecyl sulfate-polyacrylamide gel electrophoresis (SDS-PAGE). The desired protein folding was confirmed through ELISAs using the Receptor Binding Domain (RBD)-specific monoclonal antibody CR3022 (32). For detection of antibodies against NDV, whole inactivated wild type (WT) NDV virus preparations were used.

### Animal Experiments

Animal experiments were performed at an animal facility of Charles River Laboratories, Inc (PA, United States). The study was performed in accordance with the U.S. Department of Health and Human Services, Food and Drug Administration, United States Code of Federal Regulations, Title 21, Part 58: Good Laboratory Practice for Nonclinical Laboratory Studies and as accepted by Regulatory Authorities throughout the European Union (OECD Principles of Good Laboratory Practice), Japan (MHLW), and other countries that are signatories to the OECD Mutual Acceptance of Data Agreement. Nine- to ten- week old Sprague Dawley rats were used for all the experiments. Rats were co-housed in solid-bottomed cages by dose group, placing a maximum of 2 rats of the same sex and dose group per cage. Rats from the control group were housed separately, on a different rack, from the rats in the treatment group. The rats were socially housed, provided with Crink-l’ Nest™, a resting platform, and a chewing item (i.e., *ad libitum-*pelleted rodent food), except during study procedures or activities. The rats were placed on the Certified Rodent Diet^®^ #5002 (PMI^®^ Nutrition International) and fed pellets *ad libitum*, except during designated procedures.

After a period of acclimation of at least 6 days, rats were separated to perform two different vaccination experiments. A ‘main’ group, in which assessments were performed on days 1, 15, and 17 after the first vaccine dose administration, and a ‘recovery group’ in which rats were followed for a 12-day period after the administration of the second vaccine dose. In both experiments, 10 males and 10 females in each treatment group were administered 200 µL of live NDV-HXP-S vaccine intranasally (IN), intramuscularly (IM), or *via* a combination of the two routes - IN followed by IM - on days 1 and 15. Control groups received 0.9% saline solution. For the intranasal administration, each naris was sprayed twice, 30 ± 5 min apart, with 50 µL of vaccine or saline (control) for a total of 100 µL per naris per rat using a pipette with the appropriately sized tip. For intramuscular administration, 100 µL of vaccine or saline (control) were injected into the quadriceps of both hind legs for a total of 200 µL per rat. At the initial dosing, males had body weight ranging from 278 to 400 grams and females 216 to 284 grams. Blood samples (approximately 1 mL) were collected from each rat *via* the retro-orbital plexus on days 1, 15, and 17 for the ‘main group’ and days 1, 15, and 30 for the ‘recovery group’. Blood samples were centrifuged at 3,000 g for 10 min to separate the serum, then stored and transported under frozen conditions (-60 to -80°C) to the Icahn School of Medicine at Mount Sinai where the immunogenicity analyses took place.

### Toxicology Analyses

The potential toxicity and local site reactogenicity of the NDV-HXP-S vaccine were assessed to examine the vaccine’s impact on the overall health of the rats. To this end, different groups of toxicological parameters, including: a) clinical observations, b) clinical pathology, and c) post-mortem evaluations, were examined.

a) Clinical observations consisted of the visual inspection of the injection sites and the area surrounding the intranasal administration, measurements of body temperature, body weight, food intake over two different week periods, ophthalmological examinations, and mortality. For the injection site observations and intranasal evaluations, each rat was evaluated at the administration site on a minimum of four separate occasions, including the day of initial administration (before dosing), and 24 ± 1, 48 ± 1, and 72 ± 1 hours after the vaccine administration. The following score criteria were used (0): no evidence of nasal symptoms (1), nasal rattling or sneezing (2), nasal discharge on external naris, and (3) evidence of mouth breathing. If a non-zero score was recorded at the 72-hour post-dose evaluation, scoring was continued daily (every 24 ± 1 hours) until the observations were resolved or the rat was euthanized. In rats dosed intramuscularly the levels of erythema, edema, atonia, desquamation, and fissuring were scored under the following criteria (0): no symptoms (1), slight symptoms (2), moderate symptoms, or (3) severe symptoms. Scab formation was indicated with an “F” (focus/foci present) or “P” (patches present), while the presence of eschar tissue or necrotic tissue was indicated with a “N”. Ophthalmologic evaluations were performed by board-certified veterinary ophthalmologists at The Animal Eye Center of New Jersey.

b) Clinical pathology parameters consisted of hematology, coagulation, clinical chemistry, and urinalysis measurements. Before testing hematology parameters, any blood samples containing clots were discarded from the analysis. The measurements evaluated included red and white blood cell count, red blood cell distribution width, hemoglobin concentration, hematocrit count, mean corpuscular volume, mean corpuscular hemoglobin count and concentration, platelet count, absolute reticulocyte, neutrophil, lymphocyte, monocyte, eosinophil, basophil, and large unstained cell counts. The coagulation parameters were measured using plasma samples produced by transferring blood into tubes containing sodium citrate (3.2% of the total volume) and centrifuging the tube within 30 minutes of collection at room temperature for 15 minutes or more. The coagulation of the blood samples was assessed by measuring activated partial thromboplastin and prothrombin times and fibrinogen counts. Clinical chemistry parameters were tested on sera produced by first allowing the blood samples to clot for 20 to 60 minutes in serum separator tubes before being centrifuged at room temperature for at least 15 minutes. These sera were tested for measures of alanine aminotransferase, aspartate aminotransferase, alkaline phosphatase, gamma-glutamyltransferase, creatine kinase, total bilirubin, urea nitrogen, creatinine, calcium, phosphorus, total protein, albumin, calculated globulin, albumin-to-globulin ratio, glucose, cholesterol, triglycerides, sodium, potassium, and chloride. Finally, urine samples from rats were collected overnight on the day before euthanasia. These urine samples were then evaluated on their color, appearance and clarity, specific gravity, volume, pH, and levels of protein, glucose, bilirubin, ketone, and blood present.

c) Post-mortem evaluations consisted of a complete necropsy examination after rats were euthanized by exsanguination. This examination included evaluations of the carcass and musculoskeletal system, external surfaces and orifices, cranial cavity, external surfaces of the brain, and thoracic, abdominal, and pelvic cavities with their associated organs and tissues. In addition, organ weights were measured, and histological examinations of tissue specimens were performed. The tissues utilized for histology were embedded in paraffin, sectioned, mounted on glass slides, and stained with hematoxylin and eosin. Microscopic changes were assessed by microscopy and performed by a board-certified veterinary pathologist.

### Enzyme-Linked Immunosorbent Assay (ELISA)

Serological testing against SARS-CoV-2 proteins was performed using enzyme-linked immunosorbent assays as previously described (31). Briefly, 96-well polystyrene microtiter plates (Immulon 4HBX; ThermoFisher) were coated with 50 µL/well of recombinant spike (2 µg/ml) or NDV virus preparations (5 µg/ml) in phosphate-buffered saline (PBS, pH 7.4, Gibco) overnight at 4°C. Plates were washed three times with PBS and 0.1% Tween-20 (PBS-T) using an automated microtiter plate washer (AquaMax 2000; Molecular Devices). For the SARS-CoV-2 S-based ELISAs, plates were blocked with 200 µL PBS-T, 3% (v/w) nonfat dry milk (AmericanBio); for NDV-based ELISAs, PBS-T with 3% goat serum (Gibco) and 0.5% nonfat dry milk (AmericanBio) was used. After one hour of incubation, the blocking solution was removed and dilutions of rat sera were added to the plates at a 1:100 initial dilution, either in PBS-T 1% (w/v) plus non-fat dry milk (PBS-T 1% milk, AmericanBio), for the S-based ELISAs or with 3% goat serum (Gibco) and 0.5% nonfat dry milk (American Bio), for the NDV-based ELISAs. The monoclonal antibody (mAb) 6xHis (Takara) was used to detect the histidine tag present on the recombinant spike proteins used. After a two-hour incubation, plates were washed three times with PBS-T, followed by addition of 50 µL of anti-rat IgG-peroxidase antibody diluted 1:6,000 in the same solution used for serum dilutions respectively. After 1 hour of incubation at room temperature, plates were washed three times with PBS-T and incubated with 100 µL/well of *o*-phenylenediamine dihydrochloride (Sigmafast OPD, Sigma) for 10 min. The reaction was stopped by adding 50 µL per well of 3M hydrochloric acid (HCl, ThermoFisher). Optical density was measured at 490 nm using a plate reader (Synergy H1; Biotek). Analyses were performed using Microsoft Excel and Prism 9 (GraphPad). Antibody levels were expressed as area under the curve (AUC).

### Microneutralization Assay

Rat sera collected 15 days after administration of the second vaccine dose was used to assess the neutralization of SARS-CoV-2 USA‐WA1/2020 as previously described (33) with some protocol variations. All procedures were performed in a biosafety level 3 (BSL-3) facility at the Icahn School of Medicine at Mount Sinai following appropriate safety protocols. Briefly, after inactivation of rat sera (56°C for 1 hour), samples were serially diluted (threefold) from a starting dilution of 1:10. For serum dilutions, infection media consisting of minimum essential media (MEM, Gibco) supplemented with 2 mM L-glutamine, 0.1% sodium bicarbonate (w/v, Gibco), 10 mM 4-(2-hydroxyethyl)-1-piperazineethanesulfonic acid (HEPES, Gibco), 100 U/ml penicillin, 100  μg/ml streptomycin (Gibco) and 0.2% bovine serum albumin (MP Biomedicals) was used, and plates containing dilutions were stored at 4°C. Vero E6 cells were seeded onto 96-well cell culture plates at a density of 20,000 cells per well in complete Dulbecco’s Modified Eagle Medium (cDMEM). On the next day, samples were incubated with 60 tissue culture infectious dose 50% (TCID_50_) of USA-WA1/2020 for 1 hour at 37°C, 5% CO_2_ and 120μl of the sera-virus mix were transferred in a mirror-fashion to Vero-E6 plates. Sera-virus mix was removed and additional serum at the same dilution was added to the cells and plates were incubated for 48h at 37°C.

For staining of the SARS-CoV-2 nucleoprotein (NP) antigen, cells were fixed overnight at 4°C with 200 μl/well of a 10% formaldehyde solution. Plates were washed with PBS (pH 7.4) (Gibco) and permeabilized by adding 150 μl/well of PBS, 0.1% Triton X-100 for 15 min at RT. For blocking, plates were washed with PBS and incubated with PBS, 3% bovine serum albumin (BSA) for 1h at RT. During this time the mAb 1C7C7 (anti-SARS nucleoprotein antibody produced at the Center for Therapeutic Antibody Development at The Icahn School of Medicine at Mount Sinai ISMMS) was biotinylated according to the manufacturer’s instructions (Thermo Scientific EZ-Link NHS-PEG4-Biotin). Blocking solution was discarded and 100 μl/well of biotinylated mAb 1C7C7 at a concentration of 2 µg/ml in PBS, 1% BSA was added for 2h at RT. Plates were washed twice with PBS and 100 μl/well of streptavidin HRP (Thermo Scientific) diluted in PBS, 1% BSA were added at a 1:3000 dilution and plates were incubated for 1h at RT. Plates were washed twice with PBS, and 100 μl/well of OPD (Sigmafast OPD; Sigma-Aldrich) were added for 10 min at RT, followed by addition of 50 μl/well of a 3M HCl solution (Thermo Fisher Scientific). Optical density (OD) was measured (490 nm) using a microplate reader (Synergy H1; Biotek). Analysis was performed using Prism 9 software (GraphPad). After subtraction of background and calculation of the percentage of neutralization with respect to the “virus only” control, a nonlinear regression curve fit analysis was performed to calculate the inhibitory dilution 50% (ID_50_), with top and bottom constraints set to 100% and 0% respectively.

### Statistical Analysis

Analyses among the different vaccination and control groups were performed using Prism 9, (GraphPad, USA). A regular two-way analysis of variance (ANOVA) with Tukey multiple comparisons test was used. All adjusted P values of <0.05 were considered statistically significant with a confidence interval of 95%.

## Results

### Experimental Design

**Figure 1:**
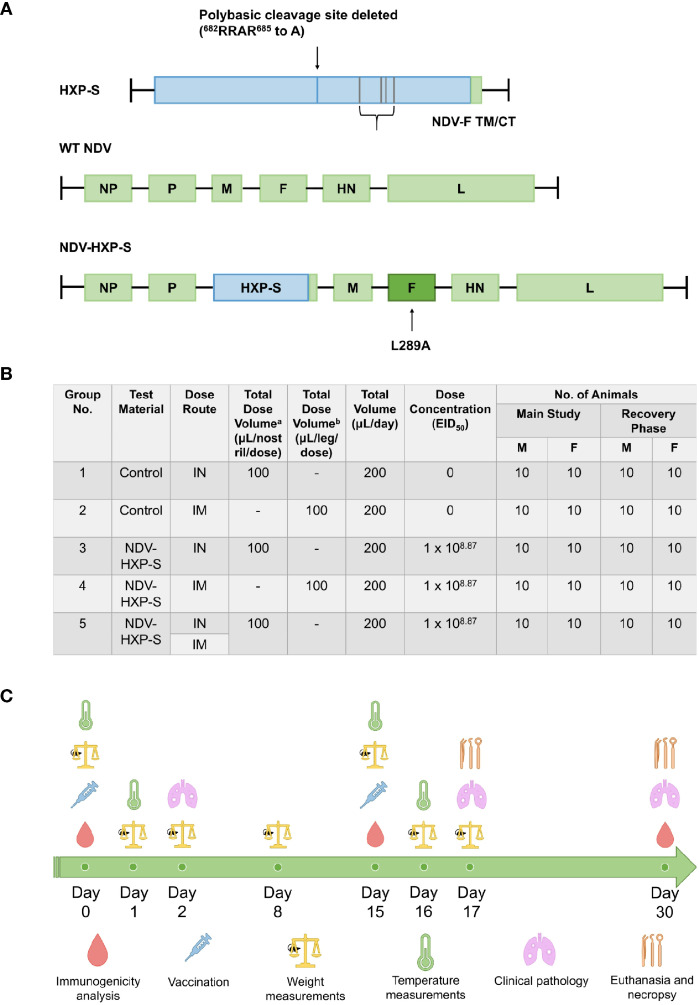
Schematic representation of NDV-HXP-S production and testing in rats. **(A)** Vector design. The S/F chimera was created by fusing the spike protein ectodomain (S), containing HexaPro stabilizing mutations, to the transmembrane domain and cytoplasmic tail (TM/CT) of the Newcastle Disease Virus (NDV) fusion (F) protein *via* a short GGGGS linker. The three arginines (R) in the polybasic cleavage site (RRAR) were removed to eliminate the cleavage site. The construct was termed HXP-S, which is codon optimized for mammalian expression. The HXP-S nucleotide sequence was incorporated between the P and M genes of the La Sota NDV genome carrying the L289A mutation in the F protein. **(B)** Groups distribution. Rats were separated into five groups: intranasal (IN) control (group 1), intramuscular (IM) control (group 2), IN vaccinated (group 3), IM vaccinated (group 4), and IN-IM vaccinated (group 5). Rats receiving the vaccine by uneven administration routes first received the intranasal dose at day 0, followed by the intramuscular dose at day 15. Intranasal dose volume was administered to each nostril twice at 50 µL/occasion, 30 ± 5 minutes apart, for the total dose volume of 100 µL/naris, or 200 µL total. Intramuscular dose volume was administered to the thigh muscles of both hind legs, for the total dose volume of 100 µL/leg, or 200 µL total. **(C)** Experimental design. All the groups received a vaccine prime at day 0 followed by a boost at day 15. Serum samples were collected for immunogenicity analyses at days 0, 15, and 30 after the first vaccine dose administration. Weight measurements were performed on days 0, 1, 2, 8, 15, 16, and 17 post-vaccination. Body temperatures (°C) were measured at 0-, 6- and 24-hours post-dosage, after both the prime and boost vaccine administrations. Clinical pathology assessments were performed on days 2, 17, and 30 following the first vaccination. Rats were euthanized on day 17 and day 30 for the main and recovery groups, respectively.

Several vaccination approaches against SARS-CoV-2 are under intensive investigation worldwide. Here, we used the previously designed NDV-HXP-S vaccine based on the NDV LaSota strain expressing a chimeric protein consisting of the spike (S) ectodomain from SARS-CoV-2 and the transmembrane domain and cytoplasmic tail (TM/CT) of the NDV fusion protein (27, 30). The codon optimized spike protein construct - for mammalian expression - named HXP-S, contained the HexaPro stabilizing mutations previously reported (34) and three arginines (R) in the polybasic cleavage site (RRAR) were removed to render it non-functional (30). Further, the HXP-S nucleotide sequence was incorporated in between the P and M genes of La Sota NDV genome carrying the L289A mutation in the F protein (**Figure 1A**). The resulting virus comprising the vaccine was expanded in embryonated eggs in the Vaccine and Cell Therapy Laboratory (VCTL) at the Icahn School of Medicine at Mount Sinai. Sprague Dawley rats 9-10 weeks old were separated into five groups: intranasal (IN) control (group 1), intramuscular (IM) control (group 2), IN vaccinated (group 3), IM vaccinated (group 4), and IN-IM vaccinated (group 5). The total number of animals included per group was 20 (10 males and 10 females) for the ‘main group.’ The same number of animals was included in the ‘recovery group’. Rats were administered a prime of the vaccine on day 0 and a boost on day 15. In group 5, animals received the intranasal dose at day 0, followed by the intramuscular dose at day 15. For the intranasal administration, rats were administered 50 µL of the vaccine to each nostril twice, 30 ± 5 minutes apart, for a total dose volume of 100 µL/naris, or 200 µL total. For the intramuscular administration, rats were injected in the thigh muscles of both hind legs, for the total dose volume of 100 µL/leg, or 200 µL total. Overall, the total dose of vaccine administered in a single day was 1x10^8.87^ 50% egg infectious dose (EID_50_) regardless of the administration route (**Figure 1B**).

Different parameters were assessed before and after vaccination to determine the safety and immunogenicity of NDV-HXP-S in Sprague Dawley rats. Immunogenicity analyses, comprised of antibody binding and neutralization assessments were performed using sera collected at days 0, 15 and 30. Clinical indicators of overall health, such as weight assessments, were performed on days 0, 1, 2, 8, 15, 16, and 17 post-vaccination, while body temperature (°C) was measured at 0-, 6- and 24-hours post-dosage, either after the prime or the boost vaccine doses. Clinical pathology assessments were performed on days 2, 17, and 30 following the first vaccination. Finally, rats were euthanized on day 17 and day 30 for the main and recovery groups, respectively (**Figure 1C**).

### Clinical Features in Vaccinated Rats

**Figure 2:**
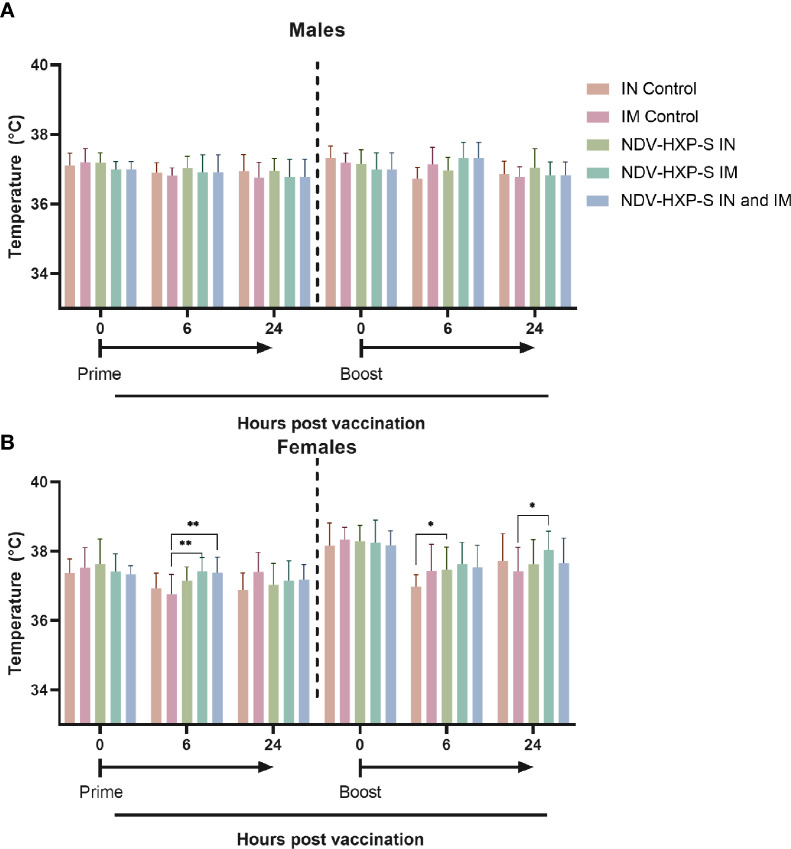
Kinetics of body temperature in vaccinated rats. Individual body temperatures (°C) were taken at 0, 6 and 24 hours post vaccination, after both prime (left side) and boost (right side) vaccine doses, in both males **(A)** and females **(B)**. Geometric mean of daily body temperature with geometric standard deviation (SD) for the IN control, IM control, IN vaccinated, IM vaccinated, and IN-IM vaccinated groups is shown. Statistically significant differences between the vaccinated groups and their respective controls are shown. The IN-IM administered group was compared with the IM control group. Statistical significance is indicated as follows: *P < 0.0413; **P < 0.0063.

As part of the parameters to evaluate the overall health of the rats after vaccination, the area close to the injection sites and the area surrounding the intranasal administration were visually inspected. There were no significant changes at the sites of the vaccine administration, including the intramuscular injection or the intranasal administrations, in either NDV-HXP-S or control groups. There were some rare cases of fur staining, thin fur, missing pinna, or skin scabs, however these were distributed across the treatment and control groups with the same frequency and there was no evidence of dose-dependent side effects. Likewise, we measured the rats body temperature, and found that administration of the vaccine, did not have a substantial impact on this parameter and no fever was detected in any of the animals. Males did not show changes in body temperature up to 24 hours post-dose (**Figure 2A**). In females, slight elevations in body temperature were observed in some of the rats, however these elevations resolved at 48 hours after dosing and were found in both NDV-HXP-S and control groups (**Figure 2B**).

**Figure 3:**
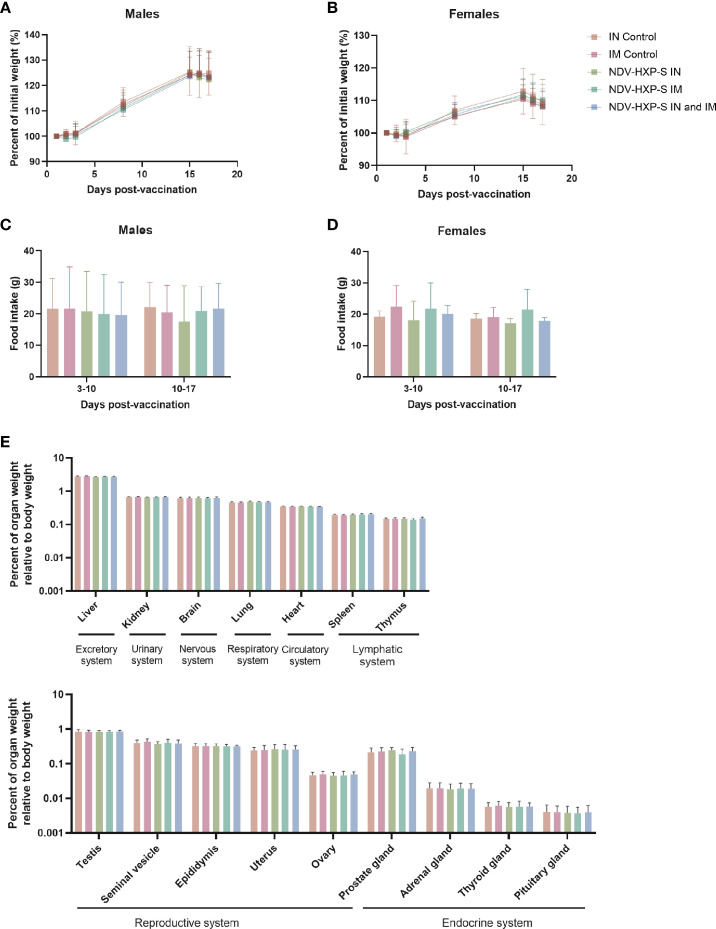
Clinical and post-mortem evaluations in vaccinated rats. The percentage of initial weight following vaccination of male **(A)** or female **(B)** rats is shown. Weight measurements were performed on days 1, 2, 3, 8, 15, 16, and 17 after vaccination. Food consumption was measured daily and averaged over two different periods: 3-10 or 10-17 days after vaccination. The average food intake in males **(C)** and females **(D)** is shown. Weight of organs corresponding to excretory, urinary, nervous, respiratory, circulatory, lymphatic, reproductive and endocrine systems are shown **(E)**. Geometric mean with geometric standard deviation (SD) for the IN control, IM control, IN vaccinated, IM vaccinated, and IN-IM vaccinated groups is shown in **(A–E)**. No significant differences between the vaccinated groups and their respective controls were detected.

We then assessed the body weight at different time points and measured the food intake during a full week in two different periods. There were no differences in group mean body weight - expressed as percentage of initial weight - among the groups administered with NDV-HXP-S and control groups, either in males (**Figure 3A**) or in females (**Figure 3B**). Likewise, there were no significant differences in food consumption between the groups administered NDV-HXP-S and the control groups (**Figures 3C, D**). In addition, all rats had normal ophthalmologic findings. Moreover, within the pathology assessments (complete list described in methods section), all parameters measured including hematology, coagulation, clinical chemistry, and urinalysis measurements, were within range, with no vaccination-induced variations or side effects observed. Finally, in terms of mortality, all rats survived until scheduled euthanasia and the rats appeared normal at necropsy. The complete necropsy examination after rats were euthanized showed normal anatomy of the carcass and musculoskeletal system, external surfaces and orifices, cranial cavity, external surfaces of the brain, and thoracic, abdominal, and pelvic cavities with their associated organs and tissues (**Supplementary Tables 1**, **2**). The weights of the organs were consistent with the control groups (**Figure 3E**), and histological examinations of the different tissues showed absence of abnormalities or tissue damage. Overall, these results recap the optimal safety profile of the NDV-HXP-S vaccine and compare well with previous findings in other animal models (27–29).

### Evaluation of the Immunogenicity of NDV-HXP-S in Rats

**Figure 4:**
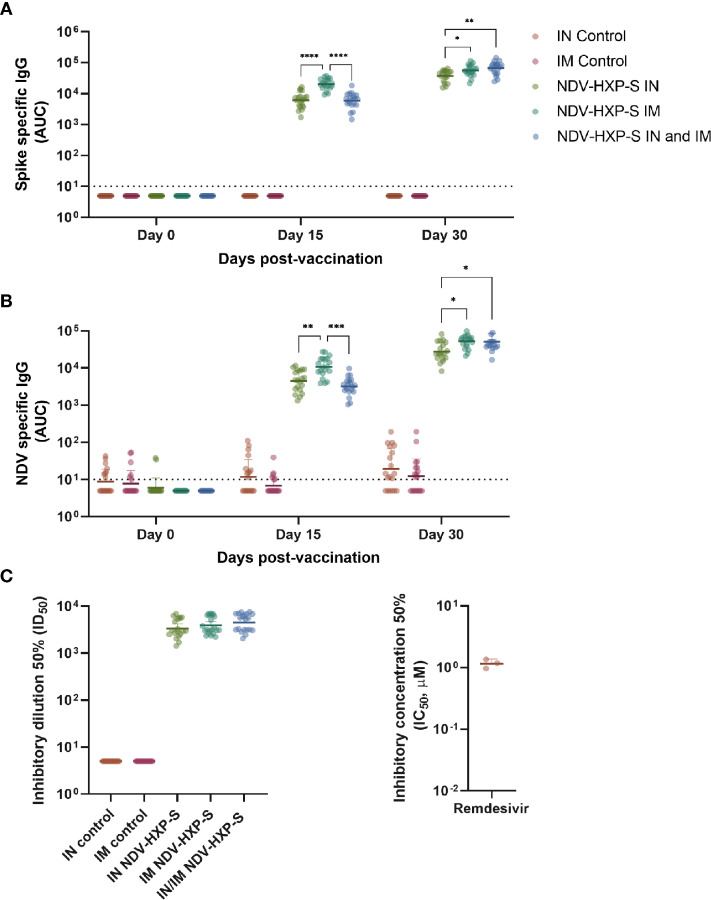
Immunogenicity of NDV-HXP-S after intranasal and intramuscular administration to rats. IgG antibody levels against wild type spike from SARS-CoV-2 **(A)** and NDV virus preps **(B)** were measured by ELISA in sera from vaccinated rats at days 0, 15 and 30 after the first vaccine dose administration. In both cases, antibody levels are expressed as area under the curve (AUC). The neutralization capacity of serum antibodies against the authentic USA-WA1/2020 SARS-CoV-2 isolate at day 30 after the first vaccine dose administration is shown in **(C)**, and titers are expressed as inhibitory dilution 50% (ID_50_, right panel). As a control, the inhibitory concentration 50% (IC_50_ - μM) of the virus polymerase inhibitor remdesivir is shown (right panel). Geometric mean with geometric standard deviation (SD) for the IN control, IM control, IN vaccinated, IM vaccinated, and IN-IM vaccinated groups is shown in all panels. In **(A–C)**, all vaccinated groups showed statistically significant differences vs their respective controls. The IN-IM administered group was compared with the IM control group. Statistically significant differences between the IN, IM and IN-IM groups are shown. Statistical significance is indicated as follows: *P ≤ 0.0270; **P ≤ 0.0032; ***P ≤ 0.002; ****P < 0.0001.

Different arms of the immune system are involved in protection against COVID-19. Particularly, the induction of humoral immunity and the presence of neutralizing antibodies have been suggested to play a major role in prevention of SARS-CoV-2 infection (8, 9), transmission (10), and development of severe disease and death (11, 12). To assess the level of antibodies induced by vaccination in Sprague Dawley rats with NDV-HXP-S, we performed ELISAs. Serum samples from rats receiving the prime (day 0) and boost (day 15) of the vaccine intranasally, intramuscularly or by a combination of IN (day 0) and IM (day 15) administration, were tested against a recombinant version of the wild type spike protein (**Figure 4A**) or against NDV preparations (**Figure 4B**). As controls, sera from rats receiving saline solution IN or IM were included in the assays. A swift increase in SARS-CoV-2 spike- or NDV- specific IgG was detected two weeks after the first vaccine dose administration. Interestingly, the primary dose of the vaccine elicited significantly higher antibody titers against both antigens in the IM administered rats versus the IN administration. However, both IM and IN primed rats that were boosted intramuscularly at day 15, displayed significantly higher antibody titers than animals administered with a complete IN vaccination regimen. While the titers in the IN primed groups were on average 3-fold lower, as compared to the IM vaccinated groups, the antibody levels were still high and they reached very high levels in the three groups after the boost (**Figure 4A**).

**Figure 5:**
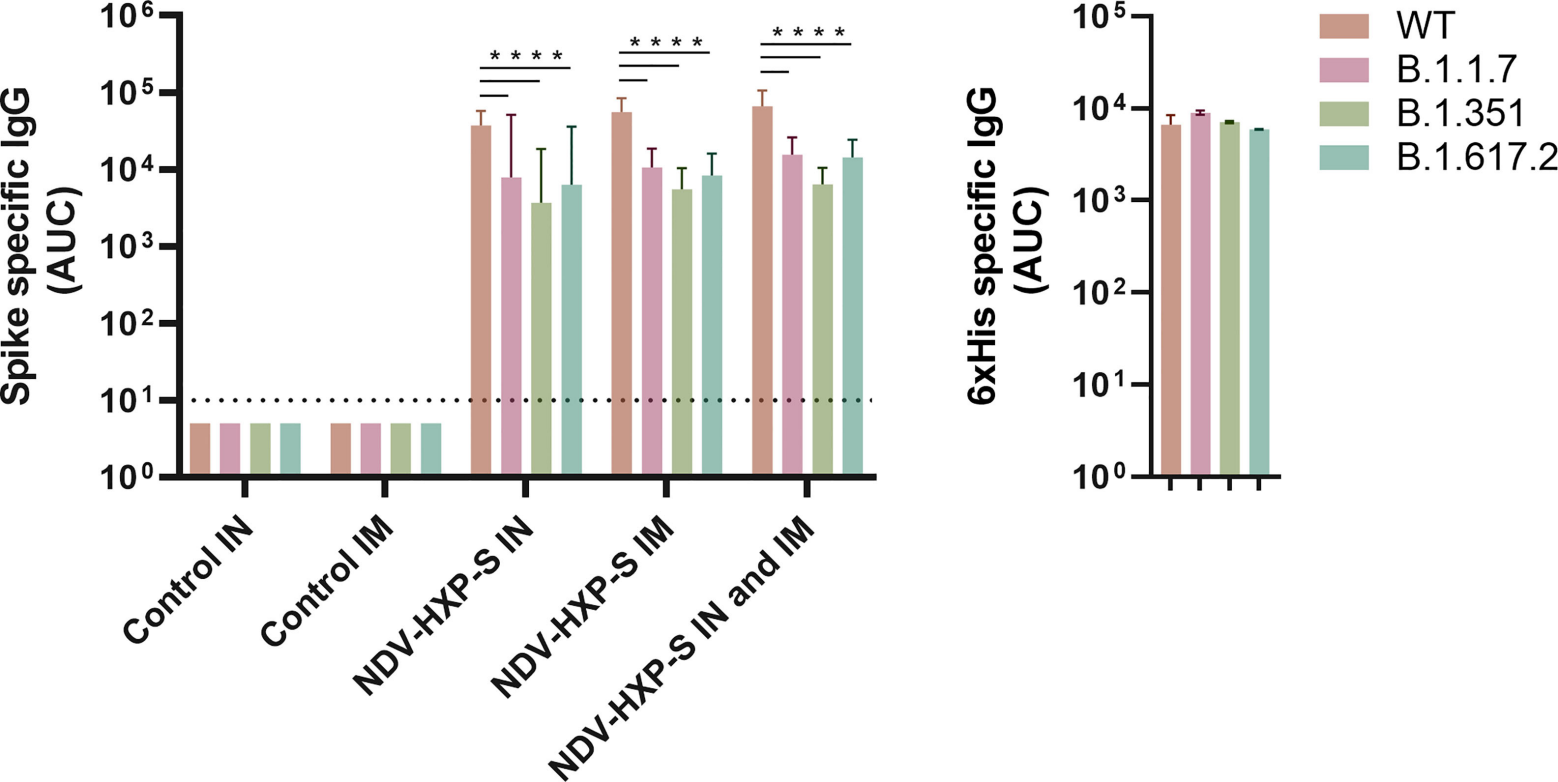
Reactivity of sera from vaccinated rats against the spike of variants of concern (VOCs). Antibody levels against the spike protein from B.1.1.7, B.1.351, and B.1.617.2 variants of concern (VOCs) and wild type (WT) spike were measured by ELISA in sera from vaccinated rats 30 days after the first vaccine dose administration (left panel). Antibody levels are expressed as area under the curve (AUC). As a control for antigen coating among the different spike proteins used, the reactivity towards the histidine tag present in all the recombinant proteins used is shown (right panel). Geometric mean with geometric standard deviation (SD) for the IN control, IM control, IN vaccinated, IM vaccinated, and IN-IM vaccinated groups is shown. Statistically significant differences between the reactivity against the spike of variants of concern (VOCs) and wild type spike are shown. Statistical significance is indicated as follows: ****P < 0.0001.

Next, to analyze the virus neutralization capacity of antibodies induced after vaccination, we tested samples from all groups, collected 30 days after the first vaccine dose administration in our standard microneutralization assay (33). Of note, our assay allows for assessment of neutralization at different stages of the replication cycle of SARS-CoV-2, since antibodies are incubated with the virus prior to the infection step and are added at the same dilution after the virus-cell incubation period. We detected excellent levels of neutralizing antibodies in all IN, IM and IN-IM groups, while control sera had no detectable neutralization in the assay (**Figure 4C**, left panel). The inhibitory concentration 50% (IC_50_) of the viral polymerase inhibitor remdesivir, is presented as a control (**Figure 4C**, right panel). Then we measured the cross-reactivity of sera from vaccinated rats against the spike protein of B.1.1.7 (Alpha), B.1.351 (Beta), and B.1.617.2 (Delta) SARS-CoV-2 VOCs. Although a significant reduction in binding was detected against the spike of the different variants tested, as compared to the wild type (WT) spike - including a deeper decline against B.1.351 -, the levels of reactivity were very high (**Figure 5**, left panel). As an antigen coating control for ELISA assays, we used an anti-6xhistidine antibody to detect the histidine tag present on the recombinant spike proteins (**Figure 5**, right panel). Overall, these data demonstrate that NDV-HXP-S is able to induce high levels of antibodies with optimal neutralizing activity either by IN or IM routes, and that these antibodies retain good reactivity against variants of concern (VOCs) of clinical importance.

## Discussion

Currently available vaccines to prevent COVID-19, although widely available in some countries, do not satisfy the existing global demand. Several regions, particularly in low- and middle-income countries, have low vaccination rates, mostly due to restricted access to vaccine doses (18, 19). Although the short-term efficacy of currently available vaccines is high, the duration of this protection is still to be determined, as well as the nature and the amplitude of the immune memory induced after vaccination. In fact, effectiveness of mRNA vaccines against infection appears to have been reduced from approximately 90% to 50%, most likely due to a combination of a decline in antibody titers and the appearance of the more infectious delta variant (35). Due to the constant emergence of SARS-CoV-2 VOCs, it is important that the vaccines introduced into the population are able to afford protection by various mechanisms, including strong humoral immunity and potent cellular responses, particularly on the mucosa of the upper respiratory tract which is the entry point of the virus (36). Vaccines that induce optimal mucosal immunity may also be better suited to block asymptomatic infection and transmission than currently used injected vaccines. Here, we evaluated the safety and immunogenicity profile of NDV-HXP-S, an NDV expressing a modified and optimized version of the spike protein of SARS-CoV-2 in rats. This novel vaccine candidate has been characterized to some extent in different preclinical models, including mice, hamsters, and pigs. A prime-boost regimen of inactivated vaccine administered intramuscularly induced high levels of spike-reactive antibodies and high virus neutralization titers *in vitro* and protected mice against challenge with a mouse-adapted strain of SARS-CoV-2 and hamsters against challenge with a wild type isolate (28). Likewise, a live version of the vaccine induced potent and protective antibody responses in mice (29) and pigs (27). Furthermore, NDV-HXP-S is currently being tested in humans in different countries including Thailand (NCT04764422), Vietnam (NCT04830800) and Brazil (NCT04993209) as an inactivated vaccine, and in Mexico (NCT04871737) as a live vaccine.

In our study, NDV-HXP-S induced robust levels of serum antibodies binding to the spike of SARS-CoV-2, regardless of the route of administration. We observed significantly lower antibody titers against the SARS-CoV-2 spike in rats receiving only one dose of the vaccine intranasally vs intramuscularly administered animals. Rats that followed a complete intranasal administration regimen vs intramuscularly administered rats or animals receiving the combined regimen, also showed significantly lower - although still high - antibody titers. Antibody titers were also detected against the NDV vector with similar trends of reactivity, which indicates that the whole viral particles are highly immunogenic. Importantly, previous studies have suggested that the NDV vector possesses adjuvant properties, which might enhance the responses against the spike protein during vaccination (37). We also determined the *in vitro* neutralization activity of antibodies contained in sera collected after the vaccine boost using our standard microneutralization assay (33). These results, showed that either of the three vaccination regimens tested elicited robust levels of neutralizing antibodies. One of the limitations of our study is that at the time when the animal study commenced, we were not able to include one of the commercial formulations as a comparator. Currently, we are testing human sera from individuals receiving NDV-HXP-S from the ongoing clinical trials and sera from individuals that received one of the commercially available vaccines. We envision that this will help to have an initial comparison about the performance of the NDV-HXP-S vaccine and other formulations. Although we were not able to assess protection after challenge with SARS-CoV-2 *in vivo*, we can anticipate that, given the amount and functionality of spike-reactive antibodies detected after vaccination, rats would likely show a robust level of protection as previously observed in mice and hamsters (28, 29). Moreover, the antibodies elicited against wild type SARS-CoV-2, displayed high levels of cross-reactivity towards the spike from other VOCs including B.1.1.7 (Alpha), B.1.351 (Beta), and B.1.617.2 (Delta). This is promising in the face of the current panorama of emergence of different VOCs worldwide.

In general, NDV-based vaccines have been shown to be safe in a wide range of hosts (37). Particularly, NDV vaccines based on the same strain that we used in this study – LaSota – have shown a good safety profile (38–40). As per recent studies, COVID-19 vaccines based on NDV expressing a wild type or a modified version of the spike protein, resulted in induction of robust immune responses, without any significant side effects in the vaccinated animals (27–29), or in humans receiving an inactivated form of the vaccine (41). To expand the safety analysis of NDV-HXP-S, we decided to examine in detail its effects on Sprague Dawley rats, which represents a standard model for toxicological analyses involving drugs, vaccines, and other biologically active and non-active compounds (42, 43). The well-characterized rat model, allows for a more detailed clinical and histopathological exploration of changes in different tissues (44–46). Here, we incorporated a more detailed analysis of pre-clinical observations, pre-clinical pathology, and post-mortem evaluation after NDV-HXP-S administration, which overall, support the safety profile of the vaccine. While we were not able to assess mucosal immunity on this study, our vaccine has demonstrated to be protective in mice and is able to induce potent antibody responses with neutralizing activity in several animal models (27–30). Induction of mucosal immunity in mice has also been demonstrated (30). Ongoing clinical trials assessing mucosal immunity after administration of the live NDV-HXP-S will inform about the strength and duration of these responses at local sites of vaccine administration. Overall, our study points out to a promising safety and immunogenicity profile of NDV-HXP-S in the population.

## Data Availability Statement

The original contributions presented in the study are included in the article/**Supplementary Material**. Further inquiries can be directed to the corresponding authors.

## Ethics Statement

Animal experiments were performed at an animal facility of Charles River Laboratories, Inc (PA, United States). The study was performed in accordance with the U.S. Department of Health and Human Services, Food and Drug Administration, United States Code of Federal Regulations, Title 21, Part 58: Good Laboratory Practice for Nonclinical Laboratory Studies and as accepted by Regulatory Authorities throughout the European Union (OECD Principles of Good Laboratory Practice), Japan (MHLW), and other countries that are signatories to the OECD Mutual Acceptance of Data Agreement.

## Author Contributions

Conceptualization, FK, PP, AG-S, WS, and JC. Investigation, JC, JT, AR, GS, HK, DB, WS, IG-D, and DS. Data curation, JT, AR, and JC. Formal analysis, JC, JT, and AR. Supervision, JC and FK. Writing - original draft, JC. Writing - review and editing: all authors. All authors contributed to the article and approved the submitted version.

## Funding

This work was mostly funded by institutional support from the Icahn School of Medicine at Mount Sinai with partial support by the Centers of Excellence for Influenza Research and Surveillance (CEIRS, contract #HHSN272201400008C) and the Centers of Excellence for Influenza Research and Response (CEIRR, contract #75N93019R00028), the Collaborative Influenza Vaccine Innovation Centers (CIVIC) contract #75N93019C00051, the JPB Foundation, the Open Philanthropy Project (research grant 2020-215611 (5384)) by anonymous donors.

## Conflict of Interest

The Icahn School of Medicine at Mount Sinai has filed patent applications relating to NDV-based SARS-CoV-2 vaccines which list WS, FK, AG-S and PP as co-inventors. FK is also listed as inventor on patent applications for SARS-CoV-2 serological assays filed by the Icahn School of Medicine at Mount Sinai. Mount Sinai has spun out a company, Kantaro, to market serological tests for SARS-CoV-2. FK has consulted for Merck and Pfizer (before 2020) as well as Goldman Sachs, and is currently consulting for Pfizer, Seqirus and Avimex. The Krammer laboratory is also collaborating with Pfizer on animal models of SARS-CoV-2. The Adolfo García-Sastre laboratory has received research support from Pfizer, Senhwa Biosciences, Kenall Manufacturing, Avimex, Johnson & Johnson, Dynavax, 7Hills Pharma, Pharmamar, ImmunityBio, Accurius, Nanocomposix, Hexamer, N-fold LLC, Model Medicines and Merck, outside of the reported work. AG-S has consulting agreements for the following companies involving cash and/or stock: Vivaldi Biosciences, Contrafect, 7Hills Pharma, Avimex, Vaxalto, Pagoda, Accurius, Esperovax, Farmak, Applied Biological Laboratories, Pharmamar, and Pfizer.

The remaining authors declare that the research was conducted in the absence of any commercial or financial relationships that could be construed as a potential conflict of interest.

## Publisher’s Note

All claims expressed in this article are solely those of the authors and do not necessarily represent those of their affiliated organizations, or those of the publisher, the editors and the reviewers. Any product that may be evaluated in this article, or claim that may be made by its manufacturer, is not guaranteed or endorsed by the publisher.
