# Supplementary material for: Safety and Immunogenicity Analysis of a Newcastle Disease Virus (NDV-HXP-S) Expressing the Spike Protein of SARS-CoV-2 in Sprague Dawley Rats
Source: Front Immunol. 2021 Nov 18;12:791764. doi: 10.3389/fimmu.2021.791764 (PMC8637447; doi:10.3389/fimmu.2021.791764)
Supplement: Supplementary file 1 [file DataSheet_1.docx]

**Supplementary table 1. Histopathology microscopic examinations after NDV-HXP-S administration to Sprague-Dawley rats (main group)**

|  | Main group (day 17 euthanasia) | | | | | | | | | |
| --- | --- | --- | --- | --- | --- | --- | --- | --- | --- | --- |
|  | Males | | | | | Females | | | | |
|  | IN control | IM control | NDV-HXP-S IN | NDV-HXP-S IM | NDV-HXP-S IN and IM | IN control | IM control | NDV-HXP-S IN | NDV-HXP-S IM | NDV-HXP-S IN and IM |
| **Nasal cavity**  Exudate  Minimal  Mild  Neutrophil exudate  Minimal  Mild  Moderate | **10**  0  0  0  0  0  0  0 | **10**  0  0  0  0  0  0  0 | **10**  0  0  0  8  3  4  1 | **10**  1  0  1  0  0  0  0 | **10**  3  0  3  0  0  0  0 | **10**  0  0  0  0  0  0  0 | **10**  0  0  0  0  0  0  0 | **10**  0  0  0  8  4  4  0 | **10**  2  1  1  1  1  0  0 | **10**  1  0  1  0  0  0  0 |
| **GALT**  Hyperplasia  Minimal | **10**  0  0 | **10**  0  0 | **10**  0  0 | **10**  0  0 | **10**  2  2 | N/A | N/A | N/A | N/A | N/A |
| **Iliac lymph node**  Hyperplasia  Minimal  Mild  Moderate  Marked | **7**  0  0  0  0  0 | **6**  0  0  0  0  0 | **7**  2  0  2  0  0 | **7**  6  2  0  2  2 | **6**  4  0  3  1  0 | **6**  0  0  0  0  0 | **6**  0  0  0  0  0 | **6**  1  0  0  1  0 | **5**  5  0  2  2  1 | **9**  6  1  3  0  2 |
| **Mandibular** **lymph node**  Hyperplasia  Minimal  Mild | **10**  0  0  0 | **10**  0  0  0 | **10**  0  0  0 | **10**  0  0  0 | **10**  2  0  2 | N/A | N/A | N/A | N/A | N/A |
| **Mesenteric lymph node**  Hyperplasia  Minimal  Mild | **10**  0  0  0 | **10**  0  0  0 | **10**  0  0  0 | **10**  0  0  0 | **10**  1  0  1 | N/A | N/A | N/A | N/A | N/A |
| **Tracheobronchial lymph node**  Hyperplasia  Minimal  Mild  Moderate  Hemorrhage  Minimal  Mild | **10**  0  0  0  0  0  0  0 | **10**  0  0  0  0  0  0  0 | **9**  2  1  1  0  0  0  0 | **9**  1  0  1  0  0  0  0 | **9**  1  1  0  0  0  0  0 | **8**  0  0  0  0  0  0  0 | **10**  0  0  0  0  0  0  0 | **9**  1  1  0  0  2  2  0 | **8**  1  0  0  1  1  0  1 | **10**  2  0  2  0  1  0  1 |
| **Skeletal muscle**  Mixed or mononuclear cell infiltration  Minimal  Degeneration  Minimal | **10**  0  0  0  0 | **9**  0  0  0  0 | **10**  0  0  0  0 | **10**  1  1  0  0 | **10**  3  3  0  0 | **10**  1  1  0  0 | **10**  0  0  0  0 | **10**  1  1  0  0 | **10**  3  3  1  1 | **10**  1  1  0  0 |
| **Sciatic nerve**  Mononuclear cell infiltration  Minimal  Mild | **10**  0  0  0 | **10**  0  0  0 | **10**  0  0  0 | **10**  5  5  0 | **10**  5  5  0 | **10**  0  0  0 | **10**  0  0  0 | **10**  0  0  0 | **10**  9  9  0 | **10**  7  6  1 |
| **Spleen**  Hyperplasia  Minimal | **10**  0  0 | **10**  0  0 | **10**  0  0 | **10**  0  0 | **10**  3  3 | **10**  0  0 | **10**  0  0 | **10**  0  0 | **10**  0  0 | **10**  3  3 |

Examinations presented were assessed 17 days post-vaccination. 10 males and 10 females were included in each group. Findings are presented by treatment group. The total number of rats examined in each group are indicated with bolded numbers. The number of rats that presented the indicated findings are shown with non-bolded numbers.

**Supplementary table 2. Histopathology microscopic examinations after NDV-HXP-S administration to Sprague-Dawley rats (recovery group)**

|  | Recovery group (day 30 euthanasia) | | | | | | | | | | | |
| --- | --- | --- | --- | --- | --- | --- | --- | --- | --- | --- | --- | --- |
|  | Males | | | | | | Females | | | | | |
|  | IN control | IM control | NDV-HXP-S IN | NDV-HXP-S IM | NDV-HXP-S IN and IM | IN control | | IM control | NDV-HXP-S IN | NDV-HXP-S IM | NDV-HXP-IN and IM |  |
| **Nasal cavity**  Exudate  Minimal  Mild  Neutrophil exudate  Minimal  Mild  Moderate | **9**  0  0  0  2  2  0  0 | **10**  0  0  0  1  1  0  0 | **10**  0  0  0  2  2  0  0 | **10**  0  0  0  0  0  0  0 | **9**  3  0  3  0  0  0  0 | **10**  0  0  0  0  0  0  0 | | **10**  0  0  0  0  0  0  0 | **10**  0  0  0  0  0  0  0 | **10**  0  0  0  0  0  0  0 | **10**  0  0  0  0  0  0  0 |  |
| **GALT**  Hyperplasia  Minimal  Mild | **10**  0  0  0 | **10**  0  0  0 | **10**  0  0  0 | **10**  0  0  0 | **10**  2  2  0 | **10**  0  0  0 | | **10**  0  0  0 | **10**  2  2  0 | **10**  3  1  2 | **10**  2  2  0 |  |
| **Iliac lymph node**  Hyperplasia  Minimal  Mild  Moderate  Marked | **10**  0  0  0  0  0 | **9**  0  0  0  0  0 | **10**  0  0  0  0  0 | **9**  4  2  1  1  0 | **7**  6  2  3  1  0 | **9**  2  2  0  0  0 | | **9**  0  0  0  0  0 | **7**  0  0  0  0  0 | **8**  6  1  5  0  0 | **8**  8  5  3  0  0 |  |
| **Mandibular** **lymph node**  Hyperplasia  Minimal  Mild | **10**  0  0  0 | **10**  0  0  0 | **10**  0  0  0 | **10**  0  0  0 | **10**  4  1  3 | **10**  0  0  0 | | **10**  0  0  0 | **10**  0  0  0 | **10**  0  0  0 | **10**  0  0  0 |  |
| **Mesenteric lymph node**  Hyperplasia  Minimal  Mild | **10**  0  0  0 | **10**  0  0  0 | **10**  1  1  0 | **10**  0  0  0 | **10**  2  1  1 | **10**  0  0  0 | | **10**  0  0  0 | **10**  0  0  0 | **10**  0  0  0 | **10**  2  0  2 |  |
| **Tracheobronchial lymph node**  Hyperplasia  Minimal  Mild  Moderate  Hemorrhage  Minimal  Mild  Moderate | **8**  0  0  0  0  0  0  0  0 | **9**  0  0  0  0  0  0  0  0 | **9**  1  1  0  0  0  0  0  0 | **9**  0  0  0  0  0  0  0  0 | **8**  4  2  2  0  0  0  0  0 | **10**  0  0  0  0  1  1  0  0 | | **8**  0  0  0  0  0  0  0  0 | **8**  0  0  0  0  1  1  0  0 | **10**  0  0  0  0  2  2  0  0 | **10**  0  0  0  0  2  0  1  1 |  |
| **Skeletal muscle**  Mixed or mononuclear cell infiltration  Minimal  Degeneration  Minimal | N/A | N/A | N/A | N/A | N/A | **10**  0  0  0  0 | | **10**  0  0  1  1 | **10**  0  0  0  0 | **10**  0  0  1  1 | **10**  0  0  2  2 |  |
| **Sciatic nerve**  Mononuclear cell infiltration  Minimal  Mild | N/A | N/A | N/A | N/A | N/A | **10**  0  0  0 | | **10**  0  0  0 | **9**  0  0  0 | **10**  0  0  0 | **10**  1  1  0 |  |
| **Spleen**  Hyperplasia  Minimal | **10**  0  0 | **10**  0  0 | **10**  0  0 | **10**  0  0 | **10**  2  2 | **10**  0  0 | | **10**  0  0 | **10**  0  0 | **10**  1  1 | **10**  0  0 |  |

Examinations presented were assessed 30 days post-vaccination. 10 males and 10 females were included in each group. Findings are presented by treatment group. The total number of rats examined in each group are indicated with bolded numbers. The number of rats that presented the indicated findings are shown with non-bolded numbers.
